# Supplementary material for: SOX21 modulates SOX2-initiated differentiation of epithelial cells in the extrapulmonary airways
Source: eLife. 2021 Jul 21;10:e57325. doi: 10.7554/eLife.57325 (PMC8331192; doi:10.7554/eLife.57325)
Supplement: Transparent reporting form [file elife-57325-transrepform.docx]

***eLife’s* transparent reporting form**

We encourage authors to provide detailed information *within their submission* to facilitate the interpretation and replication of experiments. Authors can upload supporting documentation to indicate the use of appropriate reporting guidelines for health-related research (see [EQUATOR Network](http://www.equator-network.org/%20)), life science research (see the [BioSharing Information Resource](https://biosharing.org/)), or the [ARRIVE guidelines](http://www.plosbiology.org/article/info:doi/10.1371/journal.pbio.1000412) for reporting work involving animal research. Where applicable, authors should refer to any relevant reporting standards documents in this form.

If you have any questions, please consult our Journal Policies and/or contact us: [editorial@elifesciences.org](mailto:editorial@elifesciences.org).

**Sample-size estimation**

- You should state whether an appropriate sample size was computed when the study was being designed
- You should state the statistical method of sample size computation and any required assumptions
- If no explicit power analysis was used, you should describe how you decided what sample (replicate) size (number) to use

Please outline where this information can be found within the submission (e.g., sections or figure legends), or explain why this information doesn’t apply to your submission:

In general:

All experimental procedures have been reviewed by the institutional animal welfare body. Briefly:

In order to minimize the number of animals required to achieve scientifically reliable and reproducible data, calculation of the number of animals required is based on (1) our experience of more than 20 years, (2) the expertise of colleagues in the respiratory field worldwide, (3) continuous literature survey and (4) a power analysis. The exact group sizes (numbers of dams) was calculated based on efficiency data, the necessary degrees of freedom for statistical analysis (5 pregnant dams per time point, as the embryo’s/fetuses of one pregnant dam are not entirely independent) and power analyses.

Power analysis:

Using the 2-sample T-test method with the following criteria

µ(0) = 50 (average number of cells per microscopic field in control sample)

μ(1) = 35 (average number of cells per microscopic field in treated mice, 30% difference)

σ = 10 (estimated standard deviation of the cell population)

α = 0,05 (level of significance)

P = 0,80 (power of the test

**Replicates**

- You should report how often each experiment was performed
- You should include a definition of biological versus technical replication
- The data obtained should be provided and sufficient information should be provided to indicate the number of independent biological and/or technical replicates
- If you encountered any outliers, you should describe how these were handled
- Criteria for exclusion/inclusion of data should be clearly stated
- High-throughput sequence data should be uploaded before submission, with a private link for reviewers provided (these are available from both GEO and ArrayExpress)

Please outline where this information can be found within the submission (e.g., sections or figure legends), or explain why this information doesn’t apply to your submission:

This information is provided in the figures and legends. Counting procedures and fluorescence intensity measurements are described in the material and methods. Also, the software used and the kind of tests used are described in the material and methods.

**Statistical reporting**

- Statistical analysis methods should be described and justified
- Raw data should be presented in figures whenever informative to do so (typically when N per group is less than 10)
- For each experiment, you should identify the statistical tests used, exact values of N, definitions of center, methods of multiple test correction, and dispersion and precision measures (e.g., mean, median, SD, SEM, confidence intervals; and, for the major substantive results, a measure of effect size (e.g., Pearson's r, Cohen's d)
- Report exact p-values wherever possible alongside the summary statistics and 95% confidence intervals. These should be reported for all key questions and not only when the p-value is less than 0.05.

Please outline where this information can be found within the submission (e.g., sections or figure legends), or explain why this information doesn’t apply to your submission:

This information is provided in the figures and legends. The raw data points are given in each figure. For the human airway epithelial cells each n is a different donor. This is stated in the legend. Also, for the MTEC each n is an independent mouse, which is also stated in the legend.

(For large datasets, or papers with a very large number of statistical tests, you may upload a single table file with tests, Ns, etc., with reference to sections in the manuscript.)

**Group allocation**

- Indicate how samples were allocated into experimental groups (in the case of clinical studies, please specify allocation to treatment method); if randomization was used, please also state if restricted randomization was applied
- Indicate if masking was used during group allocation, data collection and/or data analysis

Please outline where this information can be found within the submission (e.g., sections or figure legends), or explain why this information doesn’t apply to your submission:

NA

**Additional data files (“source data”)**

- We encourage you to upload relevant additional data files, such as numerical data that are represented as a graph in a figure, or as a summary table
- Where provided, these should be in the most useful format, and they can be uploaded as “Source data” files linked to a main figure or table
- Include model definition files including the full list of parameters used
- Include code used for data analysis (e.g., R, MatLab)
- Avoid stating that data files are “available upon request”

Please indicate the figures or tables for which source data files have been provided:

For each figure, source data files were uploaded
